# Divergent Genotype of Hepatitis A Virus in Alpacas, Bolivia, 2019

**DOI:** 10.3201/eid2912.231123

**Published:** 2023-12

**Authors:** Talitha Veith, L. Fabian Beltran-Saavedra, Tobias Bleicker, Marie Luisa Schmidt, José L. Mollericona, Kim Grützmacher, Rob Wallace, Jan Felix Drexler, Christian Walzer, Terry C. Jones, Christian Drosten, Victor Max Corman

**Affiliations:** Charité-Universitätsmedizin Berlin—corporate member of Freie Universität Berlin, Humboldt-Universität zu Berlin, and Berlin Institute of Health, Berlin, Germany (T. Veith, T. Bleicker, M.L. Schmidt, J.F. Drexler, T.C. Jones, C. Drosten, V.M. Corman);; Wildlife Conservation Society, La Paz, Bolivia (L.F. Beltran-Saavedra, J.L. Mollericona, R. Wallace);; Wildlife Conservation Society, New York, New York, USA (L.F. Beltran-Saavedra, J.L. Mollericona, R. Wallace, C. Walzer);; Museum für Naturkunde Berlin/Leibniz-Institute for Evolution and Biodiversity Science, Berlin (K. Grützmacher);; Deutsche Gesellschaft für Internationale Zusammenarbeit GmbH, Bonn, Germany (K. Grützmacher);; German Centre for Infection Research (DZIF), Berlin (J.F. Drexler, C. Drosten, V.M. Corman);; University of Veterinary Medicine Vienna, Vienna, Austria (C. Walzer);; University of Cambridge, Cambridge, UK (T.C. Jones);; Labor Berlin Charité–Vivantes GmbH, Berlin (V.M. Corman)

**Keywords:** hepatitis A virus, Hepatovirus, viruses, zoonoses, new-world Camelids, livestock, RNA, Bolivia, genotype, feces, alpacas

## Abstract

Hepatitis A virus (HAV) is a common human pathogen found exclusively in primates. In a molecular and serologic study of 64 alpacas in Bolivia, we detected RNA of distinct HAV in ≈9% of animals and HAV antibodies in ≈64%. Complete-genome analysis suggests a long association of HAV with alpacas.

Hepatitis A virus (HAV) causes acute hepatitis in humans worldwide; ≈159 million infections and ≈39,000 deaths were associated with HAV in 2019 (*1*), despite an available and effective vaccine (*2*). HAV infection and vaccination normally induce lifelong immunity (*2*).

The genus *Hepatovirus* (family *Picornaviridae*) consists of 9 species, designated A–I (*3*). Species B–I have been detected in small wild mammals, in seals, and in a domestic goat (*4*–*8*). HAV strains of *Hepatovirus A* have been exclusively associated with primates. All human hepatoviruses pertain to species A. Within species A, genotypes I–III are found in humans, and genotypes IV–VI are found in monkeys (*9*). No wildlife or livestock reservoir has been described for human HAV.

We screened 64 alpacas and 6 llamas in Bolivia for viruses and detected a divergent nonprimate genotype of *Hepatovirus* species A. We provide serologic evidence for a high frequency of HAV infection in New World camelids.

## The Study

**Figure 1:**
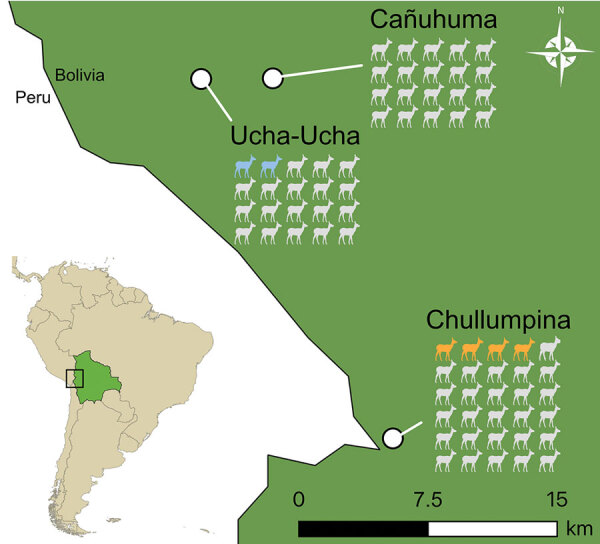
Alpaca and llama sample collection sites in study of hepatitis A virus in alpacas and llamas, Bolivia, 2019. The number of animals sampled per site is shown, 70 animals in total. Colored icons indicate alpaca HAV-positive animals by quantitative reverse transcription PCR. Inset shows locations of Bolivia and study site in South America.

We collected serum and feces samples from 64 alpacas and 6 llamas in Bolivia within the Apolobamba national protected area near the Bolivia-Peru border in 2019 (Figure 1). We tested RNA from 70 serum samples and 69 fecal samples stored in RNAlater (ThermoFisher Scientific, https://www.thermofisher.com) in pools of 8–10 using Illumina (https://www.illumina.com) high-throughput sequencing. In 3 of 16 pools, we detected matches with HAV and investigated this finding in detail.

We tested all individual samples for alpaca HAV RNA with a novel specific quantitative reverse transcription PCR (Appendix Table). Testing did not detect alpaca HAV in any of the 6 llamas. In contrast, alpaca HAV RNA was detected in 6/64 (9.4%) alpacas: in 5 of 64 (7.8%) serum samples and 5 of 63 (7.9%) feces samples. In 4 of those 6 alpacas, serum and feces samples were both positive. Concentrations of alpaca HAV RNA were up to 3.2 × 10^5^ RNA copies/mL in serum and 3.6 × 10^5^ RNA copies/mL in feces (Appendix Figure 1).

**Figure 2:**
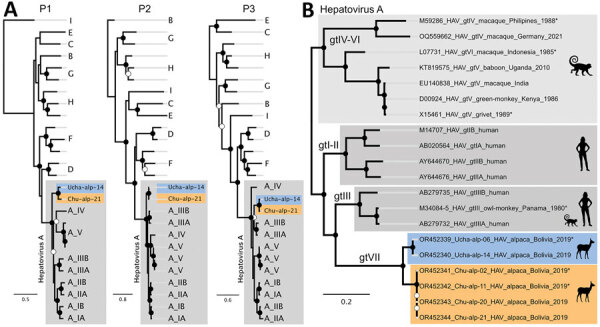
Phylogenetic analyses of alpaca HAV from Bolivia, 2019, in the context of other HAV sequences. Alpaca HAV sequences are colored by site, Ucha-Ucha in blue, Chullumpina in orange. A) Maximum-likelihood phylogenies of alpaca HAV in the context of other hepatovirus species. P1, P2, and P3 (picornavirus regions typically separated by recombination breakpoints [*10*]) nucleotide alignments were made using Clustal Omega 1.2 (http://www.clustal.org/omega) in Geneious Prime 2023.1.2 (https://www.geneious.com). ModelFinder, incorporated in IQ-TREE 1.6.12 (http://www.iqtree.org), was used to determine the best-fitting nucleotide substitution model (general time reversible model with a discrete gamma model with 4 rate categories, invariable sites, and empirical base frequencies) according to the Bayesian Information Criterion. IQ-TREE 1.6.12 was used to calculate the phylogenetic tree. The trees were rooted with *Hepatovirus* species I (P1), B (P2), and E and C (P3) respectively. Solid black circles denote ultrafast bootstrap support of >90 for the preceding branch; open circles denotes ultrafast bootstrap support between 70 and 90. GenBank accession numbers by genogroup: I, KT452658; E, KT452735; C, KT452742; B, KR703607; G, KT452730 and KT452729; H, KT452691, KT452714, and KT877158; F, KT229611, MG181943, and KT452685; D, KT452644 and KT452637; A, OQ559662, KT819575, EU140838, D00924, AB279735, AB279732, AY644670, AY644676, M14707, and AB020564. B) Maximum-likelihood phylogeny of alpaca HAV in the context of other *Hepatovirus A* genotypes. Complete genomes and partial genomes (asterisks) were used. The tree was calculated as in panel A, by using a transition model with AC=AT and CG=GT, with a discrete gamma model with 4 rate categories, invariable sites, and empirical base frequencies and rooting with *Hepatovirus* species A genotypes IV–VI. Chu-alp-11, included in this tree, tested negative in quantitative reverse transcription PCR but positive in high-throughput sequencing and pan-*Hepatovirus* PCR (Appendix Table). HAV, hepatitis A virus.

We further processed all alpaca HAV–positive samples for complete genome sequencing using undirected Illumina NextSeq sequencing (https://www.illumina.com), HAV-specific in-solution sequence capturing, and GridION reverse transcription PCR amplicon sequencing (Oxford Nanopore Technologies, https://nanoporetech.com) (Appendix Table). We generated 2 complete and 4 partial alpaca HAV genomes for further analyses (GenBank accession nos. OR452339–44). In a phylogenetic tree, alpaca HAVs form a distinct monophyletic clade within other *Hepatovirus A* sequences (Figure 2, panel A). Implied membership in the species *Hepatovirus A* is confirmed by sequence comparison. The alpaca HAV polyprotein amino acid sequence is 9%–11% distant from other *Hepatovirus A*, well within the species demarcation criterion of 30% set by the International Committee on Taxonomy of Viruses (*3*). In addition, alpaca HAV’s distance from established HAV genotypes (18%–22%) is similar to HAV genotypes’ distances from each other (14%–21%) (Table 1). Alpaca HAV regions are also similarly distant to all 3 human HAV genotypes across the genome; highest divergence is in the N terminus of 2C and in 3A (Appendix Figure 3). Therefore, alpaca HAV likely represents a distinct genotype, tentatively named genotype VII (gtVII), within *Hepatovirus A*.

**Table 1:** Nucleotide distance matrix of complete HAV genomes in alpacas, Bolivia, 2019*

Genome	Ucha-alp-14	Chu-alp-21	IA	IB	IIA	IIB	IIIA	IIIB	V
Chu-alp-21	**7.6**								
IA	18.6	19							
IB	18.6	19.1	**8.7**						
IIA	19.5	20.1	14.2	14.2					
IIB	18.7	19.2	14.1	14.2	**9.2**				
IIIA	18.2	18.6	17.3	17.3	17.3	17.2			
IIIB	18.1	18.8	17	17	17.4	17.3	**10.8**		
V	20.8	21	19.1	19	19.5	19	19.5	19.3	
VI	21.4	22	20.5	20.8	21	21	20.3	19.9	20.3

*Alpaca HAV and existing *Hepatovirus A* genotypes were included where complete genomes were available. Bold indicates intra-genotype distances. Genomes were aligned using ClustalOmega 1.2 (http://www.clustal.org/omega) in Geneious Prime 2023.1.2 (https://www.geneious.com). Nucleotide distances, given in percentages, were calculated in Geneious Prime 2023.1.2. Accession numbers used: IA, AB020564; IB, M14707; IIA, AY644670; IIB, AY644676; IIIA, AB279732; IIIB, AB279735; V, EU140838; VI, OQ559662. HAV, hepatitis A virus.

We detected alpaca HAV at 2 of 3 locations: Ucha-Ucha and Chullumpina, which are ≈20 km apart (Figure 1). The separation is reflected in phylogenetic analysis, in which 2 monophyletic clades of alpaca HAV sequences correspond to these geographic sites (Figure 2, panel B). Representative complete genome sequences from each site (Ucha-alp-14 and Chu-alp-21) are 7.6% distant in nucleotide sequence, similar to the distance between subgenotypes A and B of human genotypes I–III (≈9%–11%). Thus, alpaca HAVs from both sites could be classified into 2 subgenotypes, gtVIIA and gtVIIB (Table 1). Between both alpaca HAVs, 94.8% of nucleotide changes in the coding region are synonymous, and the dN/dS ratio, calculated with the Python dnds module (https://pypi.org/project/dnds), is 0.009, suggesting purifying selection.

Currently described HAV genotypes commonly belong to a single serotype (*11*,*12*). Neutralization epitopes are located in the capsid proteins VP1–3 (*13*), and most amino acids of those epitopes are conserved between HAV genotypes I–V and alpaca HAV (Appendix Figure 2). Thus, we were able to conduct a serologic analysis of alpaca and llama serum samples using an HAV IgG ELISA employing human HAV antigens (Mediagnost, https://mediagnost.de). Of 64 alpaca serum samples, 41 (64.1%) were reactive for HAV IgG, including the serum of 13 (81.3%) of 16 alpacas from Cañuhuma, where no alpaca tested positive for alpaca HAV RNA (Table 2; Appendix Figure 4). Of the 6 llama serum samples, 4 (66.7%) were reactive for HAV IgG (Table 2; Appendix Figure 4). In line with lifetime buildup of immunity, the proportion of seroreactive alpacas increased with age. Alpacas >2 years of age were more likely to have HAV antibodies than were younger alpacas; antibodies were present in 76.3% of older alpacas, compared with 46.2% of younger alpacas (χ2 = 6.8, d.f. = 2, n = 64; p = 0.034).

**Table 2:** Results of hepatis A virus IgG ELISA in serum of alpacas and llamas, Bolivia, 2019

Animal	No.	Reactive serum samples/total tested (%)		
		Age <2 y	Age >2 y	Total
Llamas	6	0	4/6 (66.7%)	4/6 (66.7%)
Alpacas	64	12*/26 (46.2%)	29/38 (76.3%)	41*/64 (64.1%)
Total	70	12*/26 (46.2%)	33/44 (75.0%)	45*/70 (64.3%)

*One alpaca serum sample from an alpaca <2 years of age (Chu-alp-06, positive in quantitative reverse transcription PCR) had an unclear ELISA result in 2 tests and a negative result in a 3rd test.

No sampled animal showed obvious clinical signs of a systemic or hepatic infection. However, we were not able to collect more data on the pathogenicity of HAV infection in alpacas. Other limitations of our study are the small number of samples and the limited geographic sampling range.

## Conclusions

We describe a nonprimate host association of a divergent HAV genotype in alpacas. We detected alpaca HAV RNA in both serum and feces samples, as is typically seen in acute human HAV infections. Signs of seroconversion were common, and seroreactivity increased with age. The relatively high seropositivity rate suggests that infection with alpaca HAV is common. Sequences of alpaca HAV are diversified at the nucleotide level but conserved at the amino acid level. The nucleotide diversity is consistent with a long evolutionary association of HAV with alpacas.

Hepatoviruses have been observed to undergo host-switching (*14*). A spillover event might also have been involved in HAV emergence in alpacas. However, our data are inconclusive regarding the origin of alpaca HAV and whether alpaca HAV spilled over to or from humans. More camelid and nonhuman primate HAV sequences are needed to resolve this question.

Detecting antibodies using a HAV ELISA kit with human HAV antigens suggests that alpaca HAV might belong to the same serotype as genotypes I–VI. HAV vaccination might thus provide protection from a potential alpaca HAV spillover from alpacas into humans and vice versa. Bolivia is currently considered an area of high-intermediate endemicity of HAV (*15*); increased local outbreaks and a higher burden of HAV-associated disease are expected. With that in mind, HAV vaccinations, especially for camelid handlers, should be considered to reduce spillover risk.

Appendix Additional information about divergent genotype of hepatitis A virus in alpacas, Bolivia, 2019.
