# Supplementary material for: Divergent Genotype of Hepatitis A Virus in Alpacas, Bolivia, 2019
Source: Emerg Infect Dis. 2023 Dec;29(12):2524–7. doi: 10.3201/eid2912.231123 (PMC10683824; doi:10.3201/eid2912.231123)
Supplement: Appendix — Additional information about divergent genotype of hepatitis A virus in alpacas, Bolivia, 2019. [file 23-1123-Techapp-s1.pdf]

Article DOI: https://doi.org/10.3201/eid2912.231123

EID cannot ensure accessibility for supplementary materials supplied by authors. Readers who have difficulty accessing supplementary content should contact the authors for assistance.

Divergent Genotype of Hepatitis A Virus in Alpacas, Bolivia, 2019

Appendix

Material and methods

Sampling

Sample collection from domestic new world camelids (alpacas and llamas) was conducted during routine scheduled health checks after consultation with the breeders who owned the herds, on April 16 and 17, 2019. For sample collection, animals were briefly physically restrained using established traditional methods for health checks, sampled, and then released. Fecal samples were taken from the rectum and directly transferred into cryotubes containing RNAlater (Thermo Fisher Scientific, Waltham, USA). Blood was collected from the right jugular vein, centrifuged, and serum was transferred into cryotubes. Samples were immediately cooled for 1 month and then stored at −80°C.

Nucleic acid isolation

Nucleic acid (NA) was isolated either using the QIAamp Viral RNA Mini kit (Qiagen, Hilden, Germany) or the MagNA Pure DNA and Viral NA Small volume kit (Roche Diagnostics, Basel, Switzerland) according to manufacturer’s instructions. All NA isolations of pools and individual samples used for quantitative reverse transcription PCR (RT-qPCR) were carried out using the MagNAPure system, with 100 µL input volume for serum and fecal samples, and elution in 100 µL. Repeat individual isolations were performed on QIAamp Viral RNA column, with 140 µL input volume for serum, 70 µL input volume for fecal samples, and 70 µL elution volume for serum and feces.

RT-qPCR and in-vitro transcript

Isolated nucleic acids were tested for alpaca HAV presence using specific RT-qPCR primers and probe (Appendix Table). For quantification, an alpaca HAV-specific in-vitro transcript (IVT) was designed (Appendix Table). Double stranded DNA comprising the PCR target region as well as a T7-promoter sequence was synthesized by Integrated DNA Technologies (Leuven, Belgium). In-vitro transcription was performed using the MEGAScript T7 Transcription kit (Thermo Fisher Scientific, Waltham, USA) according to manufacturer's instructions, using 100 ng of DNA as input. The 37°C incubation was carried out for 4 hours. The DNA was digested using DNase I-XT (New England Biolabs, Frankfurt am Main, Germany) according to manufacturer's instructions for 30 minutes at 37°C. The RNA was then cleaned up using the RNeasy Mini Kit (Qiagen, Hilden, Germany) according to manufacturer's instructions, with an elution volume of 50 µL. The eluate was diluted 1:10 in RNase-free water, and its RNA concentration was determined using the Qubit HS RNA kit (Thermo Fisher Scientific, Waltham, USA) according to manufacturer's instructions. The RNA was diluted to 10^9 copies/µL in water containing 10 ng/µL carrier-RNA (Qiagen, Hilden, Germany). A 1:10 dilution series in carrier-RNA containing RNase-free water was prepared. Through alpaca HAV RT-qPCR (described in detail below) of the dilution series on LightCycler 480 (Thermo Fisher Scientific, Waltham, USA), a calibration curve was created using the fit point method. The RT-qPCR was able to detect specific RNA down to 1 RNA copy/µL (equivalent to 5 copies/PCR reaction). To exclude leftover presence of DNA, a qPCR without reverse transcription was performed. IVT concentrations of 10^7 RNA copies/µL and below did not contain detectable aHAV DNA.

Isolated serum and fecal samples were tested for alpaca HAV presence and RNA concentration by RT-qPCR using SuperScript^{TM} III One-Step (Thermo Fisher Scientific, Waltham, USA). Five µL RNA was added to a mix containing 2.6 µL of RNase-free water, 12.5 µL of 2x reaction mix, 1 µL of BSA (1 mg/mL), 0.4 µL of MgSO4 (50 mM), 1 µL of each primer (10 uM), 0.5 µL of the probe (10 uM), and 1 µL of SuperScriptIII/P.Taq enzyme. Reverse transcription was performed at 55°C for 10 minutes, followed by 3 minutes of 95°C. 45 cycles of 95°C for 15 seconds and 58°C for 30 seconds were run. Quantification took place at 58°C. alpaca HAV IVT at a concentration of 10^3 copies/µL was tested in every run. Alpaca HAV RNA concentrations were determined through the created external calibration curve.

Illumina HTS and in-solution bait capturing

RNA pools, and RNA isolates in which alpaca HAV was detected, were Illumina (San Diego, USA) shotgun-sequenced. RNA concentrations were measured using the Qubit RNA HS Assay kit (Thermo Fisher Scientific, Waltham, USA). From up to 100 ng per sample, libraries were produced using the KAPA RNA HyperPrep kit (Roche Diagnostics, Basel, Switzerland) according to manufacturer's instructions. The RNA was fragmented for 6 minutes at 85°C, and the indexed libraries were PCR-amplified for 9–12 cycles. Libraries were pooled at equimolar ratio. Sequencing was performed on Illumina NextSeq (NextSeq 550 or NextSeq 1000/2000) with 150 or 300 cycles (Illumina, San Diego, USA).

To enrich alpaca HAV viral reads, a custom-designed bait-set against hepatitis viruses A-E was used. This set is based on all human hepatitis virus sequences available in the NCBI database, including HAV genotypes I-III. It contains over 77,000 individual baits (sequences available upon request), each 70 nucleotides long, and together they double-tile each input sequence. Bait sequences were collapsed so that they have a maximum nucleotide similarity of 90% for hepatitis C virus, and 97% for other hepatitis viruses. The myBaits Custom Target Capture Kit v5 was ordered from, and partially designed by Arbor Biosciences (ArborSci, Ann Arbor, USA). Capturing was performed following the manufacturer's instructions, with hybridization at 60°C and 18–22 cycles of PCR amplification.

Nanopore Sequencing

SuperScript^{TM} III One-Step (Thermo Fisher Scientific, Waltham, USA) PCRs were carried out according to manufacturer's instructions to yield amplicons for sequencing. Primer sequences can be found in the Appendix Table. Amplicons were cleaned up using KAPA Pure Beads (Roche Diagnostics, Basel, Switzerland) in a 1:1 concentration, according to manufacturer's instructions. Subsequently, libraries were prepared from the amplicons using kit SQK-PSK004 (Oxford Nanopore Technologies Limited, Oxford, UK), and multiplexing was performed using the SQK-PBK004 barcoding kit (Oxford Nanopore Technologies Limited, Oxford, UK) according to manufacturer's instructions. Libraries were sequenced on a GridION (Oxford Nanopore Technologies Limited, Oxford, UK) using the FLO-FLG001 flongle flow cell (Oxford Nanopore Technologies Limited, Oxford, UK).

ELISA

The commercial ELISA kit E10 antiHAV of Mediagnost (Reutlingen, Germany) was used to detect total antibodies against HAV. Samples were tested at a 1:10 dilution in the kit-contained dilution buffer. 100 µL of 1:10 dilution was tested according to manufacturer's instructions. The optical density (OD) was measured at 450 nm (for the reaction) and 620 nm (for background). The background OD was subtracted from the reaction OD. A blank control was included, and its OD450-OD620 was subtracted from all OD450-OD620 values, and the final value is called adjusted OD. Positive and negative controls (PC and NC respectively), as well as standards, were used undiluted. (PC+NC)/2 was used as the cutoff for positive and negative results, as suggested by the manufacturer for human samples. Samples ranging within +/− 10% of the cutoff value (termed the cutoff range) were considered to have an unclear result. All values were within the ranges expected according to the manufacturer. The ELISA used is a competition-ELISA, thus samples with adjusted OD below the cutoff are reactive, while samples with OD above the cutoff are non-reactive.

References

1. Drexler JF, Corman VM, Lukashev AN, van den Brand JMA, Gmyl AP, Brünink S, et al.; Hepatovirus Ecology Consortium. Evolutionary origins of hepatitis A virus in small mammals. Proc Natl Acad Sci U S A. 2015;112:15190–5. PubMed https://doi.org/10.1073/pnas.1516992112

2. Wang X, Ren J, Gao Q, Hu Z, Sun Y, Li X, et al. Hepatitis A virus and the origins of picornaviruses. Nature. 2015;517:85–8. PubMed https://doi.org/10.1038/nature13806

Appendix Table. Oligonucleotide sequences used in this study, including primers, probes, and in-vitro transcript

Name	Sequence 5′-3′	Comments
HAV-Alp-RTq-F	ATAGGGTAACAGCGGCGGATA	Primers and probe adapted from human HAV FDA RT-qPCR assay (https://www.fda.gov/media/160119/download)
HAV-Alp-RTq-Probe	CCTTTCAACGCCGGAGGACTGACTCTC	
HAV-Alp-RTq-R	CAATCCACTCAATGCATTCACTGG	
HAV-Alp-01-F	CCGTTTGCCTAGGCTATAGGC	Primers used to generate complete genome of Ucha-alp-14
HAV-Alp-01-R	GCTGTTACCCTATCCAAAGCATC	
HAV-Alp-02-F	CTACCAGATGACAAATGCCAGTCC	
HAV-Alp-02-R	CTGGATGTTGTAATTCCAACCTGAG	
HAV-Alp-03-F	CAGGTTGGAATTACAACATCCAGAG	
HAV-Alp-03-R	CTAGAGCGATTCTGTTCATCACTC	
HAV-Alp-04-F	GTAAATCTTGCTGATAGAATGCTGG	
HAV-Alp-04-R	CAAGTCCACTCCTTTCTGATACTG	
HAV-Alp-05-F	GGATGGATATAGTGGCCAGTTAGTG	
HAV-Alp-05-R	CACTAATAACCAGTCATCCTTGATTCC	
HAV-Alp-06-F	GGAGCTCTTGTATCTTCCAATCAG	
HAV-Alp-06-R	GCTGGTCCCATTGTCTATCAGG	
HAV-Alp-07-F	CCGTCTGGTTCTCCTTGTACG	
HAV-Alp-07-R	CACTGATCATTGAATCTCATTCTCC	
HAV-panHepato-F	GAGATAYCAYACWTATGCIAGATTTGG	Primers from (*1*) were used to generate VP2-VP3 sequence of Ucha-alp-06 and Chu-alp-11
HAV-panHepato-Rnest	CTRAATTCRTTICTCATCATYTGTG	
HAV-panHepato-R	GACATYTTIGCYCTIGCATCYTC	
HAV-Alp-IVT	TAATACGACTCACTATAGGGAGAGTAAAACGACGGCCAGTGAATGGGTGAAACCTCTTAAGCTAATACTTCTATGAAGAGATGCTTTGGATAGGGTAACAGCGGCGGATATTGGTGAGTTGTTTGACAAAAACCTTTCAACGCCGGAGGACTGACTCTCATCCAGTGAATGCATTGAGTGGATTGTATGTCAGGGCTGTCTCTAGGCTTAATTTCTGACCTCTCTGTGCGTCATAGCTGTTTCCTGTGTG	In-vitro transcript sequence includes a T7 promoter sequence, a M13 forward primer sequence, 185 bp of alpaca HAV sequence including primer and probe binding sites, and a M13 reverse primer sequence

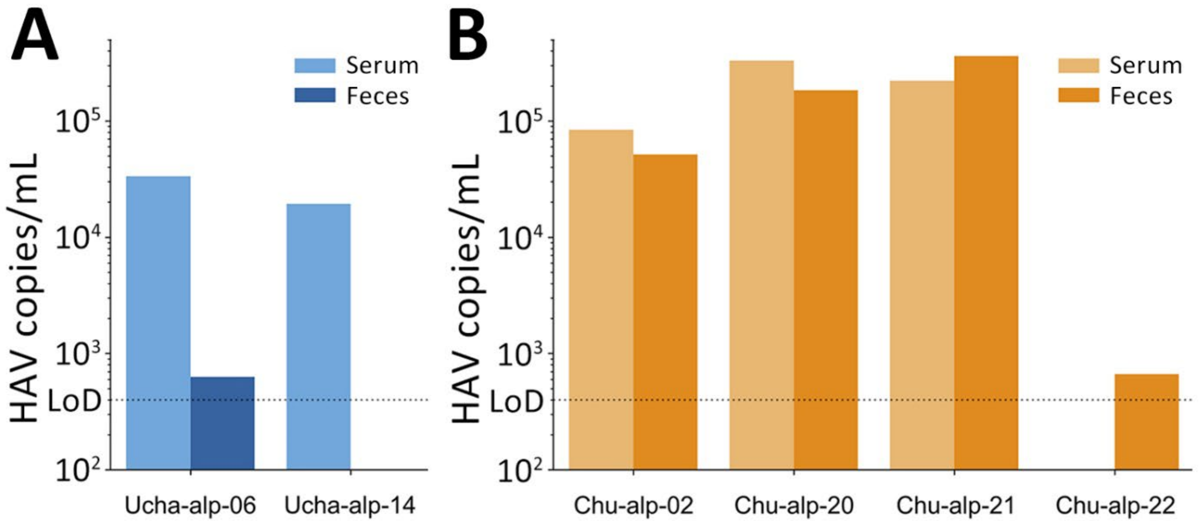

Appendix Figure 1. Alpaca HAV RNA concentration in copies/mL per site, in serum and feces samples. The lower limit of detection (LoD) is marked as a line.

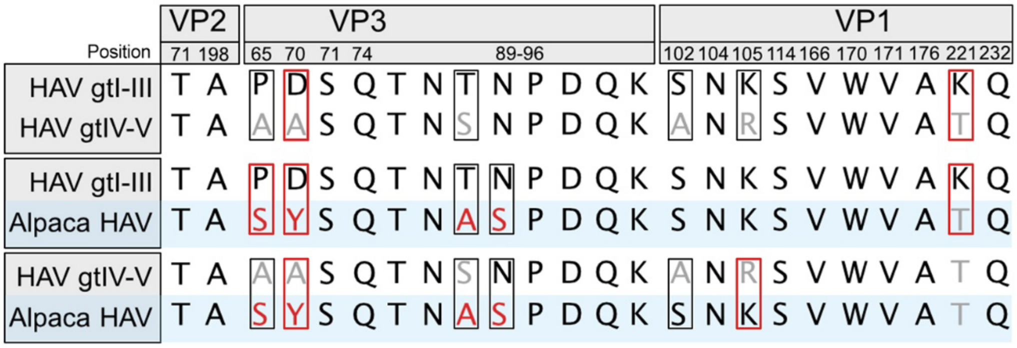

Appendix Figure 2. Comparison of Hepatovirs A genotypes in amino acids that determine the HAV neutralization phenotype (*1*,*2*). Red letters denote epitopes in which alpaca HAV differs from other HAV genotypes, gray where HAV gtIV-V differs from HAV gtI-III or where alpaca HAV has the same amino acid as HAV gtIV-V. The colors of the boxes indicate Sneath's index of amino acid difference of the two amino acids in the box (red >20, black < = 20).

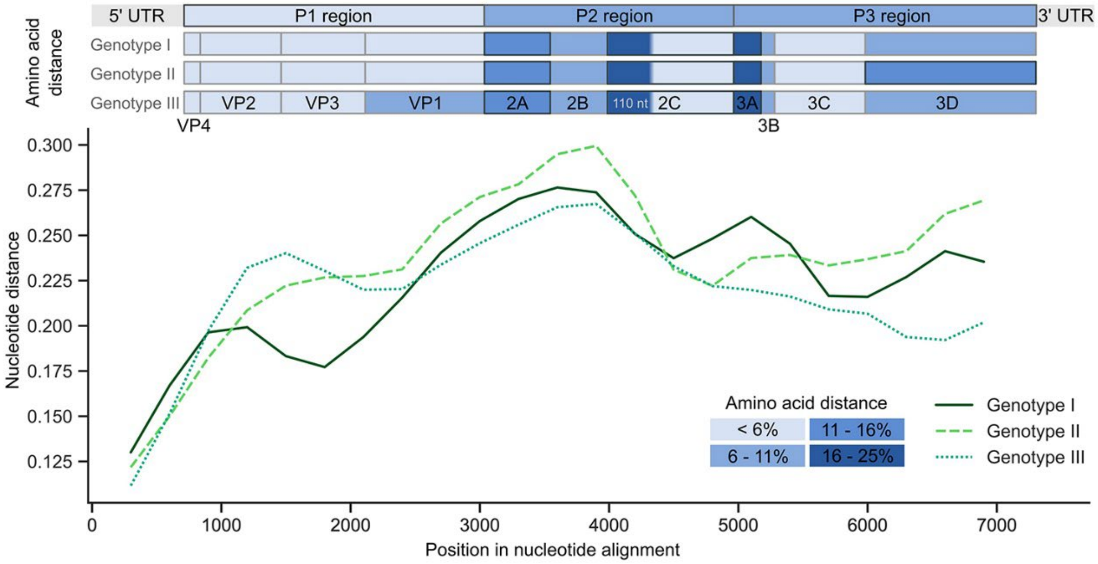

Appendix Figure 3. Alpaca HAV (Chu-alp-21) sequence comparison to human HAV genotypes I-III (AB020564, AY644670, and AB279732, respectively) across the genome on amino acid (blue) and nucleotide basis (green). The polyprotein amino acid sequences were extracted in Geneious Prime 2023.1.2, the genotype I-III sequences were pairwise aligned with alpaca HAV, and the percentage distance in all regions and open reading frames was calculated. The percentage distance describes all nonidentical sites out of the total sites. To analyze the nucleotide distance, all four sequences were multiple aligned using MAFFT 1.5.0 in Geneious Prime 2023.1.2, and patristic distances were calculated using RDP4.101, with chu-alp-21 as reference, a sliding window of 600 nucleotides and 300 nucleotides step size. The distance data was visualized with seaborn 0.12.2 within Python 3.11.4.

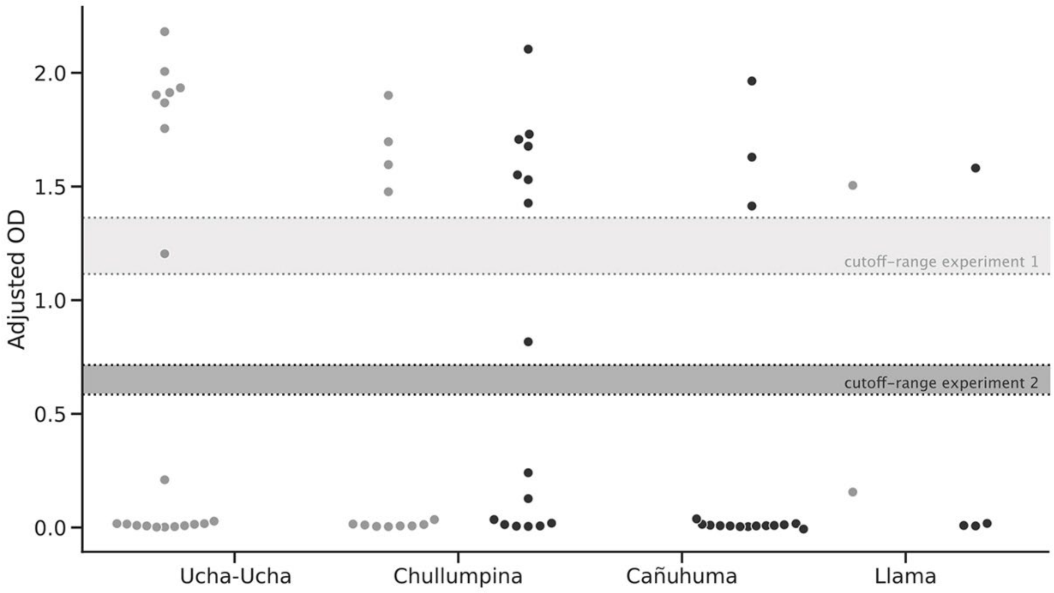

Appendix Figure 4. Raw data of HAV IgG ELISA for the 70 individual alpaca and llama serum samples. Adjusted OD represents: (sample OD450 - sample OD620) - (blank control OD450 - blank control OD620). Adjusted ODs above the cutoff-range are nonreactive, ODs below are reactive, and ODs within the range have unclear reactivity. Two separate experiments were carried out to measure all samples, shown in light and dark gray respectively. Thus, the cutoff-ranges differ between the two experiments, shown for each experiment in background color of light or dark gray. Sample Chu-alp-06 was measured in both experiments to ensure repeatability.
